# Tuberculosis Incidence Correlates with Sunshine: An Ecological 28-Year Time Series Study

**DOI:** 10.1371/journal.pone.0057752

**Published:** 2013-03-06

**Authors:** Gavin C. K. W. Koh, Gemma Hawthorne, Alice M. Turner, Heinke Kunst, Martin Dedicoat

**Affiliations:** 1 Department of Infection and Tropical Medicine, Heartlands Hospital, Birmingham, United Kingdom; 2 Department of Respiratory Medicine, Heartlands Hospital, Birmingham, United Kingdom; 3 Warwick Medical School, University of Warwick, Coventry, United Kingdom; 4 College of Medical and Dental Sciences, University of Birmingham, Birmingham, United Kingdom; McGill University, Canada

## Abstract

**Background:**

Birmingham is the largest UK city after London, and central Birmingham has an annual tuberculosis incidence of 80 per 100,000. We examined seasonality and sunlight as drivers of tuberculosis incidence. Hours of sunshine are seasonal, sunshine exposure is necessary for the production of vitamin D by the body and vitamin D plays a role in the host response to tuberculosis.

**Methods:**

We performed an ecological study that examined tuberculosis incidence in Birmingham from Dec 1981 to Nov 2009, using publicly-available data from statutory tuberculosis notifications, and related this to the seasons and hours of sunshine (UK Meteorological Office data) using unmeasured component models.

**Results:**

There were 9,739 tuberculosis cases over the study period. There was strong evidence for seasonality, with notifications being 24.1% higher in summer than winter (*p*<0.001). Winter dips in sunshine correlated with peaks in tuberculosis incidence six months later (4.7% increase in incidence for each 100 hours decrease in sunshine, *p*<0.001).

**Discussion and Conclusion:**

A potential mechanism for these associations includes decreased vitamin D levels with consequent impaired host defence arising from reduced sunshine exposure in winter. This is the longest time series of any published study and our use of statutory notifications means this data is essentially complete. We cannot, however, exclude the possibility that another factor closely correlated with the seasons, other than sunshine, is responsible. Furthermore, exposure to sunlight depends not only on total hours of sunshine but also on multiple individual factors. Our results should therefore be considered hypothesis-generating. Confirmation of a potential causal relationship between winter vitamin D deficiency and summer peaks in tuberculosis incidence would require a randomized-controlled trial of the effect of vitamin D supplementation on future tuberculosis incidence.

## Introduction

Tuberculosis is caused by *Mycobacterium (M.) tuberculosis* infection (or less commonly, *M. bovis* or *M. africanum* infection). Tuberculosis is spread by the coughing of droplets carrying live bacteria and may be pulmonary or extra-pulmonary (meningitis, osteomyelitis and lymphadenitis are common presentations). Tuberculosis is a cause of considerable morbidity and treatment involves a minimum of six months of combination antimicrobial therapy. Each year, there are an estimated 9 million new cases worldwide and tuberculosis is responsible for an estimated 1.7 million deaths [1].

Birmingham is the second largest city in the UK after London, with a population of just over one million. It has large migrant populations from Pakistan, India, Somalia and Eritrea, and 22% of the population were born outside the UK (UK Office for National Statistics, 2011 census data). Central Birmingham is endemic for tuberculosis with an annual incidence of 80 per 100,000 per year and this incidence is rising [2].

We report here the variation of tuberculosis incidence over time, looking at seasonality and sunlight as drivers of tuberculosis incidence. Exposure to sunshine is necessary for the production of vitamin D by the body and vitamin D plays an important role in the host response to tuberculosis [3]. We therefore examined the relationship between total hours of sunshine and tuberculosis notifications.

## Methods

Information was collected prospectively on all adult tuberculosis notifications (age ≥16 years) for Birmingham in the thirty-year period from 1 Jan 1980 to 31 Dec 2009, including site of infection and place of birth. Tuberculosis is a statutorily notifiable disease in the UK with notifications received from both clinicians as well as laboratory personnel. Population estimates were obtained from the UK Office for National Statistics (www.ons.gov.uk) and adjusted for the 1 April 2001 local authority boundaries changes for Birmingham. Incidence was calculated for 1982–2010 only, because adjusted mid-year population estimates were not available for years prior to 1982. Population estimates for country of birth were only available from 2004 (when the Annual Population Survey started).Historical sunshine data (hours of sunshine per month from Jan 1978 to Dec 2010) for the Shawbury weather station (37 miles from the centre of Birmingham)were obtained from the UK Meteorological Office (www.metoffice.gov.uk). Pulmonary disease was defined as pneumonia, pleural disease or mediastinal lymph node disease. All other sites were classed as extra-pulmonary. No ethical permission was required, because this study used statutorily-collected aggregate data with no identifiable patient information.

All analyses were performed on Stata 12 (StataCorp, College Station, Texas).Incidence was log-transformed (base 10) to stabilize variance. The year was divided into four seasons of three months: spring (March to May), summer (June to August), autumn (September to November) and winter (December to February) using Met Office definitions. Incomplete seasons at the start and end of the study period were omitted.

Unmeasured component models (UCMs) are commonly used in financial analysis to decompose a time series into trend and seasonal components and will also allow for the inclusion of exogenous variables. UCMs are so called because neither the trend nor the seasonal component is directly measured: instead, each is estimated from the measured (time series) data. Models may contain any, all or none of the optional components. Evidence for each of these unmeasured components comes from comparing the goodness-of-fit of models that do or do not contain the component of interest.

UCMs have been used to study seasonality in economic indicators such as monthly unemployment rates and retail indices, but have seldom been used in clinical epidemiology. We fit UCMs using maximum likelihood methods as previously described [4], [5]. We used a local level model for long term trend, *y_t_* = *τ_t_*, where *y_t_* is the tuberculosis incidence at time, *t*, and *τ_t_* is the trend component, *τ_t_* = *µ_t_+ε_t_*, and *µ_t_ = µ_t_*_–1_ *+ η_t_*. A local level model is a time series that is generated by a random walk, but with an additional noise term. For this reason, it is sometimes also known as a ‘random-walk-plus-noise model’. This, along with other models for the trend component are described in detail elsewhere [4].

Seasonality was modelled either stochastically or deterministically. The stochastic model was *y_t_* = *τ_t_*+*γ_t_+γ_t_*_–1_+ *γ_t_*_–2_+ *γ_t_*_–3_, where *γ_t_+γ_t_*_–1_+ *γ_t_*_–2_+ *γ_t_*_–3_ =  ζ*_t_*. The variables *ε_t_*, *η_t_*, and ζ*_t_*, denote independent and normally distributed random errors. The deterministic model was *y_t_* = *τ_t_*+*β*_1_ *γ*_1_+ *β*_2_ *γ*_2_+ *β*_3_ *γ*_3_, where winter is the comparator, *γ*_1_ is spring, *γ*_2_ is summer, *γ*_3_ is autumn, and *β*_1_ to *β*_3_ are the corresponding coefficients to be estimated. Deterministic models fit better than stochastic models in every instance. Analyses were stratified by site of infection (pulmonary or extra-pulmonary) and by country of birth (UK-born and non-UK-born).

The influence of sunshine on tuberculosis incidence was modelled as *y_t_* = *τ_t_*+*α_t-i_x_t–i_*_,_ where *x_t–i_* is the total hours of sunshine at time *t* with a lag of *i* seasons and *α_t-i_* is the corresponding coefficient to be estimated.

We included age and sex as potential confounders in our analysis, but in no instance did it change our results and in every instance, the models that included these factors were a worse fit than the one that did not (results not shown).

The *p*-values reported are for the relative likelihood test, the comparator being the model with the trend component only (that is, without seasonality or sunshine data). Relative likelihood was calculated using the corrected Akaike information criterion (AICc). We selected the AICc over the likelihood ratio because it rewards model fit while penalizing over-fitting and does not require models to be nested [6].

## Results

There were 9,739 cases of tuberculosis notified in the period from Dec 1981 to Nov 2009. Characteristics of those cases are in Table 1.

**Table 1:** Summary of patient characteristics.

		Frequency	%
**Age**	0–16 years	1,177	12.1
	17–64	6,982	71.7
	≥65	1,580	16.2
**Sex** *	Male	5,071	52.2
	Female	4,649	47.8
**Place of birth** **	non-UK†	1,816	69.2
	UK	807	30.8
**Site of infection** ‡	Pulmonary	6,799	69.8
	Extra-pulmonary	2,922	30.0

* Gender was missing in 19 cases.

** Last five years of study only.

† In those for whom country of birth is recorded, the five largest non-UK countries were Pakistan (35.8%), India (20.1%), Somalia (13.9%), Bangladesh (4.9%) and Zimbabwe (3.0%).

‡ 18 cases did not have site of infection notified.

**Figure 1:**
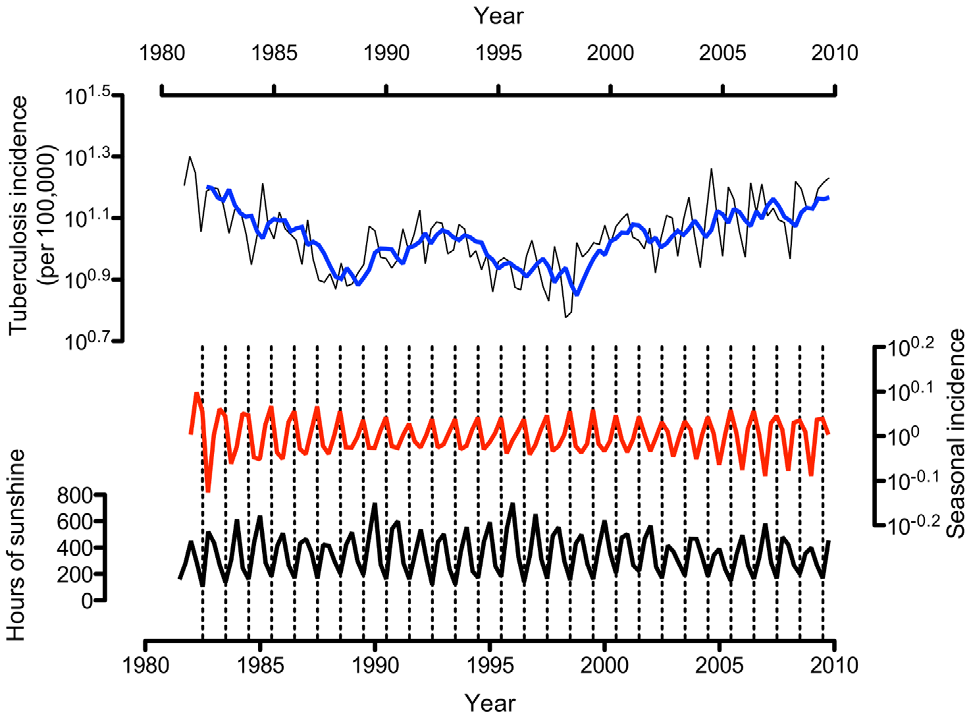
Total hours of sunshine and tuberculosis notifications. Note.– The thin black line above is the tuberculosis incidence per season. The tuberculosis incidence has been decomposed into a trend component (the thick blue line above) and a stochastic seasonal component (the thick red line across the middle of the graph). The thick black line below is the total hours of sunshine per season shifted two seasons (six months) to the right. Thin vertical interrupted lines mark the winter troughs in hours of sunlight. The graph shows that troughs in the total hours of sunshine per season correlate with peaks in the number of tuberculosis notifications two seasons later.

With the seasonal component removed, the trend in tuberculosis incidence was clearly decreasing in the first eight years of the study, but has increased steadily since 1998 (Figure 1). These trends were present in the four subgroups examined (pulmonary, extra-pulmonary, non-UK born, UK-born).

**Figure 2:**
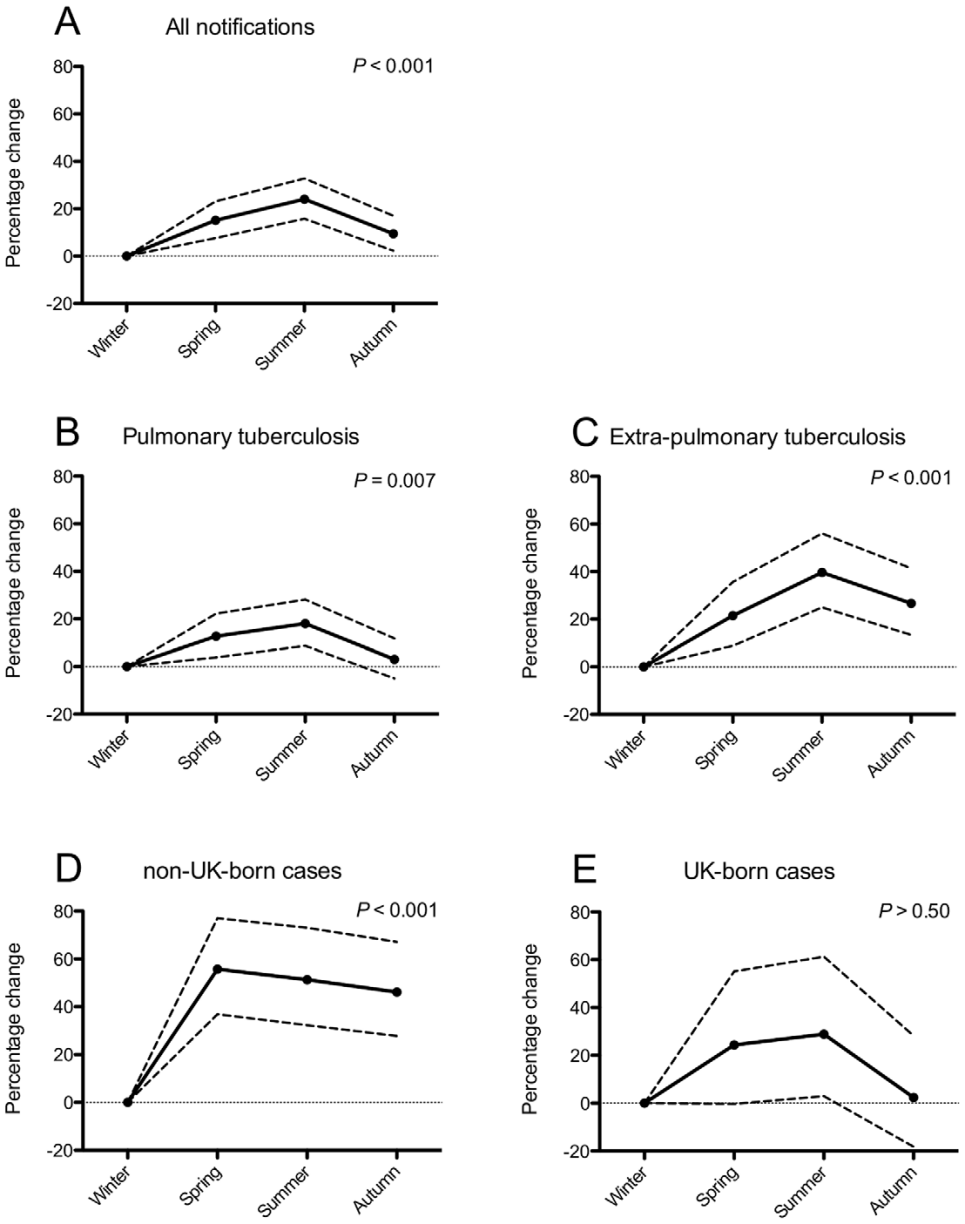
Seasonal difference in tuberculosis notifications. Note.– The solid lines show the percentage of excess notifications compared to winter, the interrupted lines are the 95% confidence intervals. **A.** all notifications; **B.** pulmonary tuberculosis only; **C.** extra-pulmonary tuberculosis only; **D.** Non-UK-born cases only; **E.** UK-born cases only.

There was good evidence for seasonality in tuberculosis incidence, with notifications 24.1% higher in summer compared to winter (95% confidence interval [95CI] 15.8–32.8%, *p*<0.001, Figure 1 and Figure 2A). Seasonality was present in both pulmonary (18.0%, 95CI 8.8–28.1%, *p* = 0.007, Figure 2B) and extra-pulmonary tuberculosis (39.6%, 95CI 24.9–56.1%, *p*<0.001, Figure 2C). There strong evidence for seasonality in non-UK born cases (51.3%, 95CI 32.3–73.1%, *p*<0.001, Figure 2D), but although a summer-time peak was seen in UK-born cases (28.9%, 95CI 3.0–61.3%, Figure 2E), there was no statistical evidence to support this (*p*>0.50).

Median total hours of sunshine per season was 346 (interquartile range 214 to 466 hours), ranging from a low of 166 (157 to 182) hours in winter to 523 (448 to 593) hours in summer). Total hours of sunshine was inversely correlated with total tuberculosis notifications two seasons (six months) later (4.7% increase in cases for each 100 hours decrease in sunshine, Figure 1, *p*<0.001).No relationship was found for lags of one or three seasons. Low levels of sunshine were correlated with high numbers of notifications two seasons later for both pulmonary (4.0%, *p* = 0.003) and extra-pulmonary tuberculosis (5.5%, *p*<0.001).There was strong statistical evidence for a relationship between hours of sunshine and tuberculosis incidence in non-UK-born cases (11.7%, *p*<0.001) but there was no statistical support for a relationship in UK-born cases (7.8%, *p*>0.50).

## Discussion and Conclusion

Tuberculosis incidence in Birmingham fell during the 1980s, rose again at the end of the 1990s and continues to rise. This has been ascribed primarily to the influx of migrants to the UK from countries where TB is highly endemic [7].

We found that tuberculosis notifications in Birmingham were seasonal, with peaks occurring annually during the summer (June to August). This pattern was seen in every subgroup examined, but the small number of UK-born cases meant that power was reduced, confidence intervals were wide and results failed to reach statistical significance.

We proceeded to examine the relationship between hours of sunshine and tuberculosis notifications, and found that low levels of sunshine in winter were associated with high numbers of tuberculosis notifications in the summer (Figure 1). This pattern was present in all subgroups, except UK-born cases. Again, this lack of statistical evidence for an association may be due to lower power in this subgroup rather than a true lack of association.

Seasonality of tuberculosis notifications has been described previously [8]–[11] and has been known since before the advent of antimicrobial chemotherapy [12]. Douglas and others previously reported a lack of seasonality in UK-born cases [13], however, this contradicts both historical (pre-chemotherapy era) [12] and national data [8], both of which show evidence of seasonality even in white Caucasian populations. Although we found no statistical evidence for seasonality in UK-born cases, inspection of Figure 2E suggests a seasonal pattern that is not detected only because of a lack of statistical power. Specifically, the point estimates for summer in the UK-born subgroup were similar to those in the whole study population (28.9% versus 24.1%) and the confidence intervals for the whole study population (Figure 2A) are entirely contained within those for the UK-born subgroup (Figure 2E). This means that a seasonal trend in UK-born cases cannot be excluded by this study and larger studies are needed.

*Mycobacterium tuberculosis* is a slowly dividing organism (every 15 to 20 hours) [1], and tuberculosis has a long incubation period ranging from months to years [14]. It is therefore plausible that winter conditions may explain the summer peak in notification.

There are two major mechanisms which may drive seasonality in tuberculosis. First, winter crowding may lead to increased transmission of tuberculosis, which then manifests as active tuberculosis in the summer. Second, reduced exposure to sunshine in the winter and decreased vitamin D levels may result in impaired host defence to tuberculosis. The potential link between sunlight and vitamin D levels is interesting, because vitamin D supplementation of at-risk populations is a plausible public health intervention.

Vitamin D is a steroid hormone synthesized in the presence of sunlight and vitamin D levels are lower in winter [9]. It is required for gamma interferon-mediated macrophage responses, which play a critical role in the host response to *M. tuberculosis* infection [15], and triggers the synthesis of antimicrobial peptides such as cathelicidin and defensins [16], [17]. A systematic review has linked strongly vitamin D deficiency to tuberculosis prevalence [18].

Despite this, use of vitamin D supplements in active disease has not proven successful, except in those carrying polymorphisms in their vitamin D receptor [3]. A pilot study in children with latent tuberculosis suggests that it may still be a useful treatment for preventing progression of latent tuberculosis [19], although no large clinical trials have been carried out to date. Our study is the first to report the link between sunshine and tuberculosis incidence in adults, and extends the work of Visser and others, who studied only meningitis in children [20].

Our study has a number of strengths. We report 28 years of data, which is the longest time series of any published study. As we used statutory notifications from both clinical and laboratory sources, we believe this data to be essentially complete.

Our study has a number of limitations. First, population data by country-of-birth was not available for the whole study period, which means we were able to calculate tuberculosis incidence within the UK-born and non-UK-born subgroups for the period 2004 to 2009 only, exacerbating power issues in the UK-born subgroup. Second, we cannot exclude the possibility that another factor closely correlated with the seasons is responsible. For instance, travel to home countries where TB is endemic, could plausibly vary by season. Our findings should therefore be considered hypothesis generating. Third, statutory notifications do not include information about the length of time in the UK and socioeconomic status, and these could not therefore be accounted for in the analysis. Fourth, pro-vitamin D3 synthesis is dependent on the UVB (290–315 nm) portion of the solar spectrum, however, UVB exposure does not correlate perfectly with total hours of sunshine [21].

Lastly, exposure to sunlight depends not only on total hours of sunshine but also on other factors [22] that will vary according to each individual. The amount and type of clothing worn is dependent on ethnic, cultural and religious factors. For example, more clothes will be worn in cold weather, and fewer clothes in warm weather. Birmingham has a large Pakistani population which is primarily Muslim, and Muslim women are often heavily covered outdoors for religious and cultural reasons, and this is not dependent on time of year. Individuals may use sun screen or hats in sunny weather so as to reduce the risk of sun burn and to prevent skin cancer. Individuals may choose to stay indoors in order to avoid cold or hot weather, or may be obliged to work indoors or outdoors for much of the day because of their occupation. These individual factors are not accounted for in this analysis.

Confirmation of a potential causal relationship between winter vitamin D deficiency and summer peaks in tuberculosis incidence would require a large longitudinal study in a population with high tuberculosis incidence. It is difficult to justify leaving proven vitamin D deficiency untreated, and such a study would therefore have to be conducted as a randomized-controlled trial (RCT) of the effect of vitamin D supplementation on future tuberculosis incidence in the whole population, or an RCT of vitamin D as an adjunct to other treatment for latent tuberculosis.

In conclusion, tuberculosis incidence in Birmingham was found to be seasonal, with peaks occurring every summer that relate inversely to total hours of sunlight six months earlier. We suggest that reduced exposure to sunlight in the winter may result in lower vitamin D levels and higher susceptibility to tuberculosis.
